# Cell-laden alginate dialdehyde–gelatin hydrogels formed in 3D printed sacrificial gel

**DOI:** 10.1007/s10856-020-06369-7

**Published:** 2020-03-09

**Authors:** Dalia Dranseikiene, Stefan Schrüfer, Dirk W. Schubert, Supachai Reakasame, Aldo R. Boccaccini

**Affiliations:** 1 Institute of Biomaterials, University of Erlangen-Nuremberg, 91058 Erlangen, Germany; 2 Institute for Polymer Materials, University of Erlangen-Nuremberg, 91058 Erlangen, Germany

## Abstract

Alginate dialdehyde–gelatin (ADA–GEL) hydrogels have been reported to be suitable matrices for cell encapsulation. In general, application of ADA–GEL as bioink has been limited to planar structures due to its low viscosity. In this work, ring shaped constructs of ADA–GEL hydrogel were fabricated by casting the hydrogel into sacrificial molds which were 3D printed from 9% methylcellulose and 5% gelatin. Dissolution of the supporting structure was observed during the 1^st^ week of sample incubation. In addition, the effect of different crosslinkers (Ba^2+^ and Ca^2+^) on the physicochemical properties of ADA–GEL and on the behavior of encapsulated MG-63 cells was investigated. It was found that Ba^2+^ crosslinked network had more than twice higher storage modulus, and mass decrease to 70% during incubation compared to 42% in case of hydrogels crosslinked with Ca^2+^. In addition, faster increase in cell viability during incubation and earlier cell network formation were observed after Ba^2+^ crosslinking. No negative effects on cell activity due to the use of sacrificial materials were observed. The approach presented here could be further developed for cell-laden ADA–GEL bioink printing into complex 3D structures.

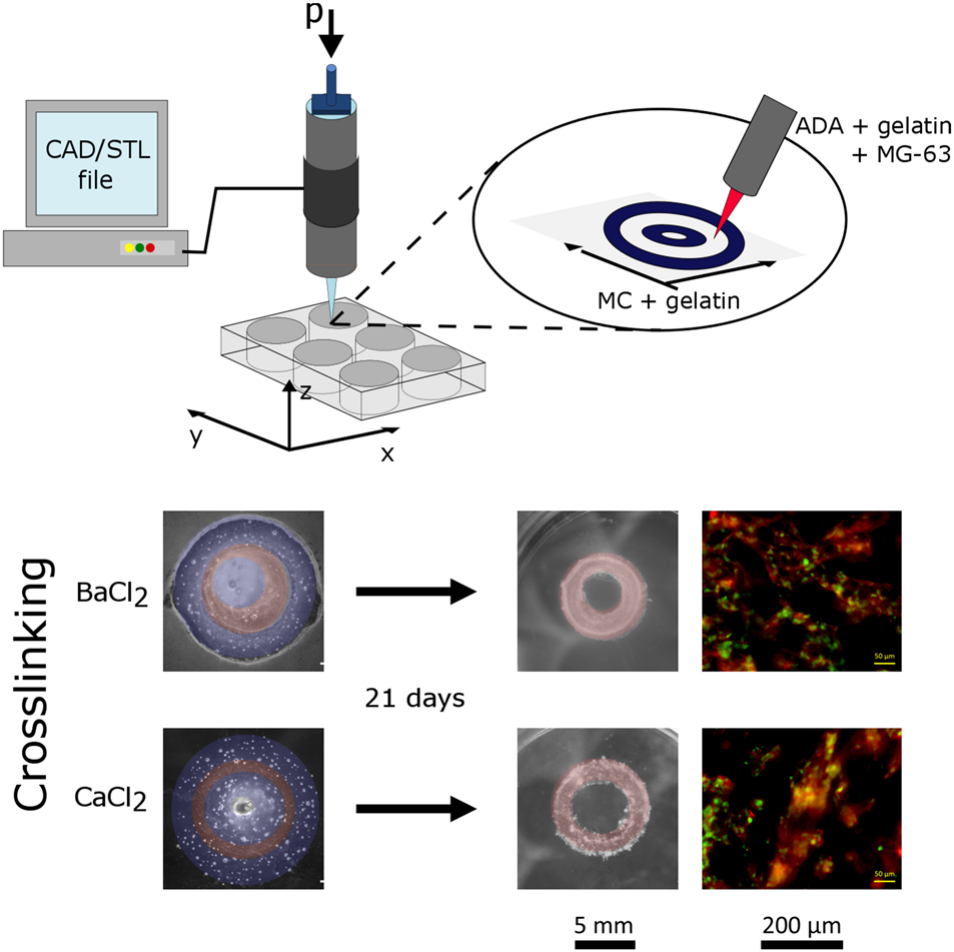

## Introduction

Biofabrication encompasses processing techniques that allow to create 3D structures of cell-laden hydrogels for tissue engineering applications [1]. Different strategies have been used in order to provide mechanical support to 3D printed constructs like depositing bioinks into a liquid bath [2, 3] or the co-printing of hard [4] and soft [5] supporting materials. Methylcellulose (MC) hydrogels have also been tested as a sacrificial material for 3D printing [6].

Alginate dialdehyde–gelatin (ADA–GEL) hydrogel has been used for cell encapsulation and it has been shown to exhibit good cell adhesion, proliferation and migration properties [7–9]. The benefit of using such hydrogel is that two advantages—mild ADA crosslinking with divalent ions and cell adhesion to GEL—can be combined as the two components (ADA and GEL) form a covalent bond via Schiff’s base reaction [9, 10]. In this work, 3D printed sacrificial MC based structures were used for casting cell-laden ADA–GEL hydrogel matrices. The possibility to use Ba^2+^ ions for ADA crosslinking instead of Ca^2+^ in order to improve the mechanical stability of the constructs was proposed, following recent previous results on similar alginate based hydrogels [5]. In vitro cell studies were performed in order to preliminary assess the potential of the presented biofabrication approach for tissue engineering applications.

## Materials and methods

### ADA–GEL hydrogel

#### Hydrogel preparation

ADA was synthesized from sodium alginate (MW 100,000–200,000 g/mol, Sigma-Aldrich, USA) following the process reported by Zehnder et al. [8]. According to previous work of our group, this process results in a degree of oxidation of ADA of approx. 30% [10]. To prepare ADA–GEL, equal volumes of filtered 5% (w/v) ADA and 5% (w/v) GEL Type A (300 Bloom, Sigma, USA) solutions were stirred together for 10 min. The mixture was then casted, crosslinked by covering with 0.1 M CaCl_2_ (VWR, Belgium) or 0.1 M BaCl_2_ (Merck KGaA, Germany) for 15 min, and washed three times with Hank’s balanced salt solution (HBSS, Sigma-Aldrich, USA).

#### Physicochemical properties

Mechanical properties of ADA–GEL were determined by using disc-shaped samples (*n* = 3, 16 mm of diameter and thickness of approximately 1 mm) subjected to a frequency sweep in compressive deformation mode to determine storage (E′) and loss (E″) moduli at room temperature by DMTA. A suitable pre-load (40 g) and strain amplitude (0.1%) were determined by previous amplitude sweeps.

Degradation of ADA–GEL was evaluated by incubating ADA–GEL disks in Dulbecco’s modified Eagle’s medium (DMEM, Gibco, Germany) supplemented with 10% (v/v) fetal bovine serum and 1% (v/v) penicillin-streptomycin (both Sigma-Aldrich, Germany) (the same DMEM was used for cell growth) under cell culture conditions (37 °C, 95% relative humidity, 5% CO_2_). Mass change was calculated at defined time points. In parallel, the chemical composition of samples which were incubated for 7, 14, 21, 28 days was investigated by using attenuated total reflection Fourier-transform infrared spectroscopy (ATR FTIR) (IRAffinity-1S, Shimadzu, Japan). The medium was changed three times a week following the same procedure used for cell culture studies.

### Cell encapsulation in ADA–GEL ring structures

#### Sample preparation

Sacrificial gel containing 9% (w/v) MC (Sigma, USA) and 5% (w/v) GEL was used. This particular composition of the sacrificial gel was obtained from the printing tests of different gel formulations consisting of various concentrations of MC and GEL (data not shown here). Sacrificial gel was transferred into an autoclaved cartridge with a conical nozzle (G22, Nordson EFD, Germany). The cartridge was then placed in a 3D printer (BioScaffolder GeSiM 2.1, GeSiM, Germany). The sacrificial gel was printed as two concentric rings with diameters of 4.4 and 10 mm. The formed well between them was then filled with 70 μl ADA–GEL containing osteosarcoma cells MG-63 (Sigma-Aldrich, Germany) at the concentration of 1 × 10^6^ cells/ml. The samples were crosslinked by covering with CaCl_2_ or BaCl_2_ and washed three times with HBSS. The ring-shaped constructs were afterwards incubated in DMEM for 21 days under cell culture conditions.

#### Cell activity monitoring

Cell viability was monitored by using the WST-8 assay kit (Sigma-Aldrich, Germany) which was applied according to the manufacturer’s protocol. SYTOX™ green nucleic acid stain and rhodamine phalloidin (both Invitrogen™, Molecular Probes® by Life Technologies™, USA) were used for staining cell nuclei and actin filaments, respectively, for fluorescence microscopy (Axio Observer Scope D1, Carl Zeiss AG, Germany).

### Statistical analysis

Statistical analyses were performed by one-way analysis of variance (ANOVA) with Bonferroni means comparison.

## Results and discussion

### ADA–GEL hydrogel properties

**Fig. 1:**
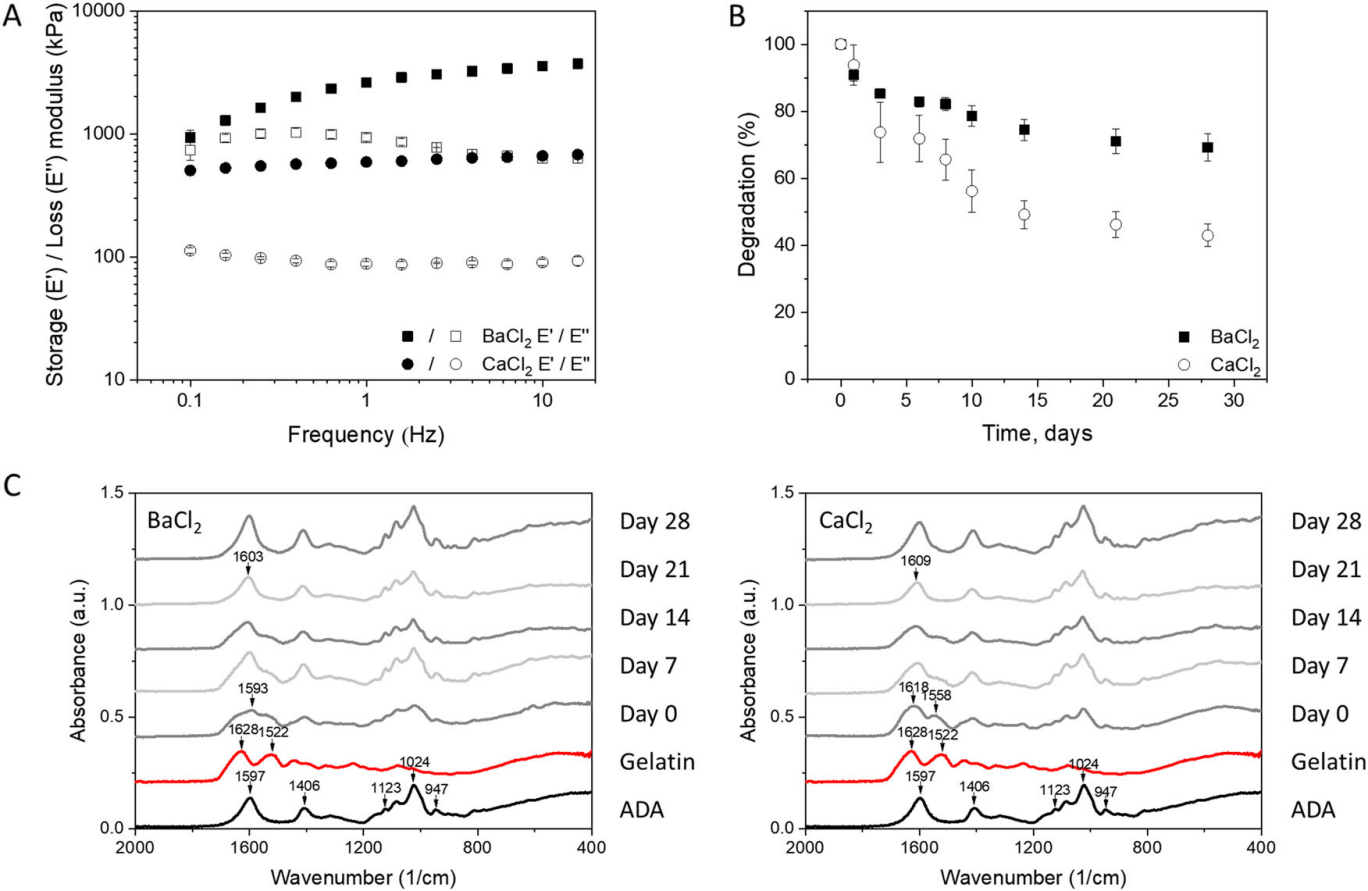
Properties of 2.5% ADA + 2.5% GEL samples crosslinked with BaCl_2_ and CaCl_2_: **a** DMTA measurements showing storage and loss moduli; **b** Degradation in DMEM over a time period of 28 days; **c** FTIR spectra obtained at different time points of incubation in DMEM (the relevant peaks are discussed in the text)

DMTA measurements showed higher mechanical properties of BaCl_2_ crosslinked samples in comparison to CaCl_2_ crosslinked samples (Fig. 1a). Moreover, storage modulus (E′) was found to be greater than the loss modulus (E″) for both sample groups showing dominating elastic behavior. The E′ increased with frequency indicating the viscoelastic behavior of the materials [9]. Small standard deviation between the measurements (5–6%) suggests that the sample crosslinking reached saturation and was reproducible.

Sample degradation (mass loss) is shown in Fig. 1b. In the case of BaCl_2_ crosslinking, the sample mass rapidly decreased to 85 ± 1% during the first 3 days and then decreased further until a final value of 69 ± 4% at 28 days of incubation. In case of CaCl_2_ crosslinking, a higher weight loss was detected. In the first 3 days the sample mass already decreased to 74 ± 9% and was further reduced to a final value of 42 ± 3% at day 28. The main difference between the two samples is given by the contribution of ADA dissolution to the overall degradation. CaCl_2_ crosslinked samples tend to loosen due to weaker bonds and higher exchange of ions with Na^+^ from the medium [11] that can result in the loss of the crosslinks that form the ADA network. Therefore, ADA molecules may be released together with GEL. The degradation of BaCl_2_ crosslinked samples is then mainly related to the release of uncrosslinked GEL.

FTIR spectra of each gel component were examined (Fig. 1c). Two peaks at 1628 and 1522 cm^−1^ of the GEL spectrum indicate amide I and amide II peaks [10]. For ADA, typical peaks of asymmetric (at 1597 cm^−1^) and symmetric (at 1406 cm^−1^) COO^−^ vibration, C–O stretching at 947 cm^−1^, C–C stretching at 1123 cm^−1^ and C–O–C stretching at 1024 cm^−1^ were found [12, 13]. Samples after mixing and crosslinking exhibited specific peaks. In case of BaCl_2_ crosslinking, a broad peak in the range 1630–1530 cm^−1^ was formed with distinguishable high peak at 1593 cm^−1^ and shoulder on the right side (1560–1543 cm^−1^). In case of CaCl_2_ crosslinking, a broad double peak at 1618 and 1558 cm^−1^ was found for the sample before incubation. With both crosslinkers, this peak was the result of overlapping peaks of amide I and the ones that correspond to the formation of Schiff’s base bond due to crosslinking between ADA and GEL [9]. The peak remained for the first 14 days of incubation and became narrower on day 21 and shifted to 1603 cm^−1^ for BaCl_2_ and to 1609 cm^−1^ for CaCl_2_. This indicates that gelatin was being released during the incubation.

### Hydrogel casting with sacrificial structure

**Fig. 2:**
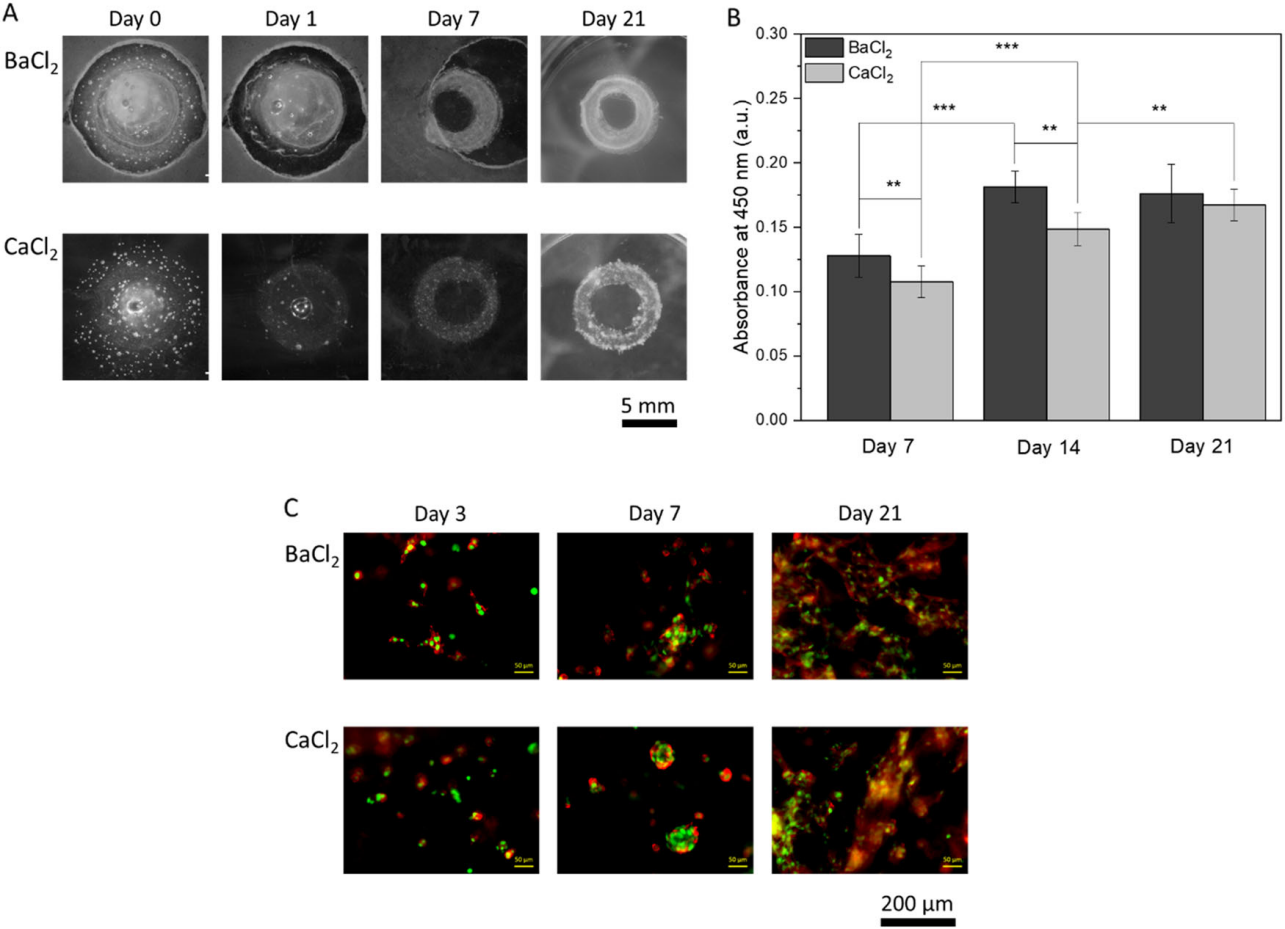
ADA–GEL ring samples formed with sacrificial hydrogel: **a** Light microscopy images; **b** Change in cell viability during the incubation period; **c** Fluorescence microscopy images

Samples containing two sacrificial rings and cell-laden hydrogel were successfully prepared (Fig. 2a). Full dissolution of the sacrificial gel was observed upon 7 days without additional swelling or disruption of the ADA–GEL ring structure. After measuring the development of cell viability (Fig. 2b), it was noticed that during the first 2 weeks of incubation the values significantly increased for both types of samples. In addition, samples crosslinked with BaCl_2_ had significantly higher values of cell viability than the samples crosslinked with CaCl_2_. After the 3rd week only in the case of CaCl_2_ crosslinked sample cell viability significantly increased further and reached a comparable value to the one of BaCl_2_ crosslinked samples. This result was also noticed when analyzing fluorescence images (Fig. 2c). Cells in BaCl_2_ crosslinked samples started to spread and formed an extended network already after 2 weeks, while cells in CaCl_2_ crosslinked samples grew in more globular structures which connected with each other during the second half of the incubation period. Such growth pattern in case of CaCl_2_ crosslinking has been previously reported by Zehnder et al. [8]. It has been shown in literature that alginate scaffolds crosslinked with sufficient amount of BaCl_2_ for 2 min, in addition to CaCl_2_ crosslinking, exhibited cell viability of over 88% 11 days post-printing [2]. The authors attributed such result to the possibility of maintaining the mechanical properties of the gel rather than to the type of ions used. The anchoring density that may be higher in BaCl_2_ crosslinked samples might also influence cell attachment [14]. The increase of cell viability with time in case of CaCl_2_ crosslinking could be due to the ECM forming molecules secreted in the gel by the cells themselves [15]. The present results thus show that the combination of sacrificial structures and a suitable cross-linking process of the ADA–GEL bioink represents a suitable approach for the biofabrication of 3D structures, namely ring forms (Fig. 2a) as well as grid structures depicted in Fig. S1 (Supplementary information).

## Conclusions

The formation of alginate dialdehyde–gelatin cell-laden hydrogels into ring-shaped structures by using 3D printed methylcellulose based sacrificial materials was successfully achieved in this work. Slower increase in cell viability was identified in samples crosslinked with CaCl_2_ in comparison to those crosslinked with BaCl_2_. This result could be attributed to differences in gel stiffness as samples crosslinked with BaCl_2_ exhibited higher elastic moduli and lower weight loss during the incubation period. Further studies considering different alginate dialdehyde–gelatin crosslinking methods could help to understand the factors defining the different cell growth patterns observed in this study. Moreover, the use of a 3D printed sacrificial gel as introduced in this work could be transferred to other materials like collagen that gels at 37 °C in longer periods of time.

## Supplementary information

Fig. S1
