# Supplementary figures and images for: Cell-laden alginate dialdehyde–gelatin hydrogels formed in 3D printed sacrificial gel
Source: J Mater Sci Mater Med. 2020 Mar 9;31(3):31. doi: 10.1007/s10856-020-06369-7 (PMC7062650; doi:10.1007/s10856-020-06369-7)

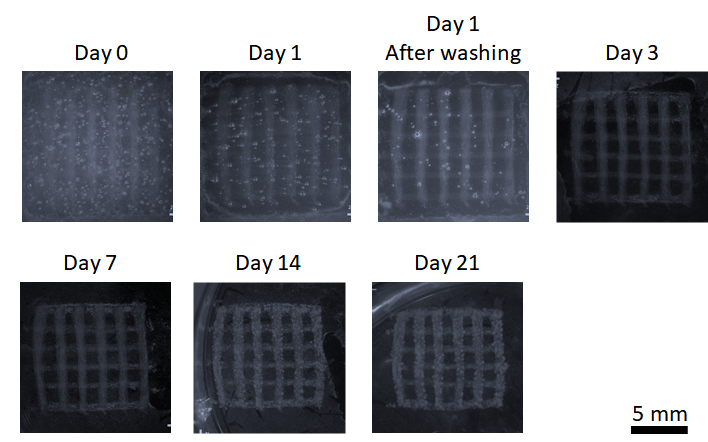

Supplement: Supplementary file 1 — Fig. S1 [file 10856_2020_6369_MOESM1_ESM.png]
